# Supplementary material for: Excess risk and clusters of symptoms after COVID-19 in a large Norwegian cohort
Source: Eur J Epidemiol. 2022 Feb 25;37(5):539–48. doi: 10.1007/s10654-022-00847-8 (PMC8872922; doi:10.1007/s10654-022-00847-8)
Supplement: Supplementary file 1 — Supplementary file1 (DOCX 207 kb) [file 10654_2022_847_MOESM1_ESM.docx]

**ONLINE SUPPLEMENTARY MATERIAL**

European Journal of Epidemiology

# **Excess risk and clusters of symptoms after COVID-19 in a large Norwegian cohort**

Ida Henriette Caspersen^1^ (ORCID: 0000-0003-2591-8435), Per Magnus^1^, Lill Trogstad^2^

^1^Centre for Fertility and Health, Norwegian Institute of Public Health, Oslo, Norway

^2^Department of Method Development and Analysis, Norwegian Institute of Public Health, Oslo, Norway

Correspondence to: Ida Henriette Caspersen (ida.henriette.caspersen@fhi.no)

**Supplementary Table 1. List of symptoms reported by all cohort participants independent of COVID-19 status.**

| **CHECK OFF IF YOU HAVE ANY OF THE FOLLOWING CONDITIONS/ AILMENTS NOW:** |
| --- |
| Exhaustion («fatigue») |
| Fever coming and going |
| Shortness of breath |
| Cough |
| Reduced lung function |
| Joint pain |
| Muscle pain |
| Chest pain |
| Fast pulse or palpitations |
| Inflammation of the heart muscle ("myocarditis") |
| Kidney disease |
| Dizziness |
| Difficulty thinking or concentrating ("brain fog") |
| Poor memory |
| Mood swings |
| Sleep problems |
| Headache |
| Depression |
| Anxiety |
| Skin rash |
| Hair loss |
| Altered smell or taste |
| Other |

**Supplementary Table 2. Loadings of three extracted factors of an exploratory factor analysis of post-acute symptoms among COVID-19 cases (n=774). The first three factors with oblimin rotation explained 55% of the total variation. The analysis includes symptoms with increased risk among COVID-19 cases 11-12 months after diagnosis.**^a^

|  | OBLIMIN rotation | | |  | VARIMAX rotation | | |
| --- | --- | --- | --- | --- | --- | --- | --- |
|  | Factor1 | Factor2 | Factor3 |  | Factor1 | Factor2 | Factor3 |
| Brain fog | 0.95 | -0.10 | 0.04 | Brain fog | 0.83 | 0.17 | 0.35 |
| Poor memory | 0.82 | 0.09 | 0.03 | Poor memory | 0.77 | 0.31 | 0.34 |
| Anxiety | 0.54 | 0.15 | -0.16 | Fatigue | 0.54 | 0.49 | 0.41 |
| Skin rash | 0.47 | 0.01 | 0.00 | Heart palpitations | 0.53 | 0.25 | 0.50 |
| Heart palpitations | 0.43 | 0.13 | 0.34 | Anxiety | 0.47 | 0.28 | 0.07 |
| Fatigue | 0.41 | 0.39 | 0.19 | Skin rash | 0.42 | 0.14 | 0.17 |
| Altered smell or taste | 0.31 | 0.10 | 0.00 | Headache | 0.36 | 0.34 | 0.55 |
| Cough | 0.14 | 0.61 | -0.28 | Dizziness | 0.35 | 0.06 | 0.93 |
| Headache | 0.14 | 0.30 | 0.46 | Altered smell or taste | 0.30 | 0.18 | 0.13 |
| Dizziness | 0.04 | 0.00 | 0.97 | Shortness of breath | 0.27 | 0.87 | 0.23 |
| Chest pain | 0.04 | 0.52 | 0.14 | Chest pain | 0.23 | 0.51 | 0.25 |
| Shortness of breath | -0.01 | 0.93 | 0.05 | Cough | 0.21 | 0.60 | -0.09 |
| Reduced lung function | -0.06 | 0.60 | 0.07 | Reduced lung function | 0.14 | 0.55 | 0.17 |

^a^ We omitted rare occurrences (myocarditis, kidney disease) and symptoms with no increased risk (based on adjusted RR) among COVID-19 cases 11-12 months after infection (depression, mood swings, sleep problems, joint pain, muscle pain, fever, and hair loss).

**Supplementary Table 3. Loadings of two extracted factors from an exploratory factor analysis of post-acute symptoms among COVID-19 cases (n=774), using varimax rotation. The analysis includes symptoms with increased risk among COVID-19 cases 11-12 months after diagnosis. The two factors explained 31% and 19% (in total 50%) of the variance in symptoms.** ^a^

|  | VARIMAX rotation | |
| --- | --- | --- |
|  | Factor1 | Factor2 |
| Brain fog | 0.87 | 0.19 |
| Poor memory | 0.80 | 0.34 |
| Dizziness | 0.74 | 0.15 |
| Heart palpitations | 0.72 | 0.28 |
| Fatigue | 0.68 | 0.48 |
| Headache | 0.60 | 0.35 |
| Anxiety | 0.44 | 0.27 |
| Skin rash | 0.44 | 0.16 |
| Altered smell or taste | 0.34 | 0.17 |
| Chest pain | 0.33 | 0.49 |
| Shortness of breath | 0.31 | 0.93 |
| Reduced lung function | 0.19 | 0.55 |
| Cough | 0.14 | 0.56 |

^a^ We omitted rare occurrences (myocarditis, kidney disease) and symptoms with no increased risk (based on adjusted RR) among COVID-19 cases 11-12 months after infection (depression, mood swings, sleep problems, joint pain, muscle pain, fever, and hair loss).

**Supplementary Table 4. Risks, excess risks (risk difference, RD) and adjusted relative risks (RR) for reporting current symptoms among cohort participants who acquired a COVID-19 diagnosis 11-12 months ago and controls with no COVID-19.**

|  | **Complete case data** | | **Imputed data** | |
| --- | --- | --- | --- | --- |
|  | **n ^a^** | **RR (95% CI), minimal adjustment ^b^** | **n ^a^** | **RR (95% CI),**  **full adjustment ^c^** |
| CIRCULATION/RESPIRATION |  |  |  |  |
| Chest pain | 63009 | 4.2 (1.5, 8.9) | 73512 | 7.0 (3.7, 13.3) |
| Cough | 62764 | 2.5 (1.2, 4.6) | 73231 | 2.3 (1.2, 4.6) |
| Dyspnea | 62786 | 8.9 (5.4, 13.3) | 73257 | 9.4 (6.1, 14.3) |
| Heart palpitations | 62322 | 4.5 (2.6, 7.2) | 72735 | 4.0 (2.4, 6.8) |
| Myocarditis | 63183 | NA | 73718 | 0 (0, 0) |
| Reduced lung function | 62823 | 20.0 (9.7, 35.5) | 73311 | 25.9 (15, 44.8) |
| BRAIN |  |  |  |  |
| Anxiety | 62065 | 3.2 (1.3, 6.3) | 72401 | 2.9 (1.3, 6.2) |
| Brain fog | 61262 | 3.1 (1.9, 4.6) | 71516 | 3.2 (2.2, 4.8) |
| Depression | 62042 | 1.7 (0.7, 3.3) | 72364 | 1.6 (0.8, 3.2) |
| Dizziness | 62237 | 1.6 (0.7, 3.1) | 72652 | 2.1 (1.1, 3.8) |
| Fatigue | 60765 | 5.1 (3.5, 6.9) | 70956 | 4.9 (3.6, 6.8) |
| Headache | 60583 | 1.7 (1.0, 2.5) | 70742 | 1.8 (1.2, 2.7) |
| Mood swings | 62186 | 1.3 (0.7, 2.2) | 72571 | 1.3 (0.7, 2.2) |
| Poor memory | 61318 | 5.2 (3.6, 7.2) | 71578 | 5.4 (3.9, 7.5) |
| Sleep problems | 59698 | 1.5 (0.9, 2.4) | 69702 | 1.5 (0.9, 2.4) |
| JOINT AND MUSCLE |  |  |  |  |
| Joint pain | 59584 | 1.9 (0.8, 3.6) | 69583 | 1.7 (0.8, 3.6) |
| Muscle pain | 59646 | 1.9 (1.0, 3.2) | 69655 | 1.7 (0.9, 3.1) |
| OTHER |  |  |  |  |
| Altered smell or taste | 73052 | 52.3 (34.9, 74.1) | 73655 | 52.4 (36.6, 74.9) |
| Fever | 63065 | 1.5 (0.1, 6.6) | 73580 | 2.8 (0.7, 11.3) |
| Hair loss | 72667 | NA | 73268 | 1.1 (0.2, 8.1) |
| Kidney disease | 63021 | NA | 73537 | NA |
| Skin rash | 62377 | 2.2 (0.8, 4.7) | 72784 | 2.4 (1.1, 5.2) |

^a^ Total number included in regression models.

^b^ Adjusted for age and chronic illness.

**^c^** Adjusted for age, chronic illness, BMI, education, and smoking.

**Supplementary Table 5. Risks, excess risks (risk difference, RD) and relative risks (RR) for reporting current symptoms for male versus female COVID-19 cases. Complete case analyses.**^a^

|  | n ^b^ | Male COVID-19 cases, n (%) with symptoms | Female COVID-19 cases, n (%) with symptoms | RD | RR (95% CI), adjusted^a, c^ |
| --- | --- | --- | --- | --- | --- |
| CARDIORESPIRATORY |  |  |  |  |  |
| Chest pain | 655 | 17 (5.2) | 23 (5.2) | 0 | 0.9 (0.5, 1.7) |
| Cough | 652 | 19 (5.9) | 32 (7.2) | 1.3 | 1.2 (0.6, 2.2) |
| Dyspnea | 655 | 37 (11.4) | 72 (16.2) | 4.8 | 1.3 (0.9, 2.0) |
| Heart palpitations | 654 | 16 (4.9) | 60 (13.7) | 8.8 | 2.9 (1.6, 5.5) |
| Myocarditis | 760 | 0 (0) | 1 (0.2) | 0.2 | NA |
| Reduced lung function | 657 | 22 (6.8) | 24 (5.4) | -1.4 | 0.9 (0.4, 1.7) |
| BRAIN |  |  |  |  |  |
| Anxiety | 646 | 9 (2.8) | 13 (3) | 0.2 | 1.3 (0.5, 3.2) |
| Brain fog | 644 | 28 (8.8) | 77 (17.7) | 8.9 | 2.0 (1.3, 3.2) |
| Depression | 648 | 11 (3.4) | 21 (4.8) | 1.4 | 1.4 (0.7, 3.0) |
| Dizziness | 648 | 17 (5.3) | 55 (12.6) | 7.3 | 3.0 (1.5, 6.1) |
| Fatigue | 639 | 52 (16.4) | 95 (22) | 5.6 | 1.2 (0.9, 1.7) |
| Headache | 641 | 31 (9.7) | 79 (18.2) | 8.5 | 1.6 (1.1, 2.5) |
| Mood swings | 653 | 19 (5.9) | 31 (7) | 1.1 | 0.9 (0.5, 1.6) |
| Poor memory | 642 | 39 (12.1) | 76 (17.6) | 5.5 | 1.2 (0.8, 1.9) |
| Sleep problems | 622 | 23 (7.4) | 55 (13) | 5.6 | 1.7 (1.0, 2.9) |
| JOINT AND MUSCLE |  |  |  |  |  |
| Joint pain | 624 | 18 (5.6) | 30 (7.2) | 1.6 | 1.5 (0.8, 2.9) |
| Muscle pain | 627 | 16 (5) | 41 (9.8) | 4.8 | 1.7 (0.9, 3.1) |
| OTHER |  |  |  |  |  |
| Altered smell or taste | 656 | 45 (13.9) | 112 (25.2) | 11.3 | 1.7 (1.2, 2.4) |
| Fever | 660 | 3 (0.9) | 7 (1.6) | 0.7 | 2.4 (0.5, 11.5) |
| Hair loss | 655 | 3 (0.9) | 15 (3.4) | 2.5 | 2.3 (0.7, 8.2) |
| Kidney disease | 759 | 0 (0) | 0 (0) | 0 | NA |
| Skin rash | 659 | 7 (2.2) | 21 (4.7) | 2.5 | 4.1 (1.2, 14.1) |

^a^ Complete cases analyses, excluding between n=97 and n=103 observations in the different regression models due to missing data in covariates.

^b^ Total number included in regression models.

^c^ Adjusted for age, chronic illness, and severity of initial infection (mild vs. moderate/severe/hospitalized).

**Supplementary Table 6. Number of symptoms reported in March 2021 among COVID-19 cases in wave 1, wave 2, and among subjects not receiving a COVID-19 diagnosis. ^a^**

|  | **COVID-19 cases, wave 1  (n=170) 11-12 months after infection** ^a^ | | **COVID-19 cases, wave 2  (n=583), 1-6 months after infection** ^a^ | | **No COVID-19  (n=73727), symptoms emerging last 12 months** ^b^ | |
| --- | --- | --- | --- | --- | --- | --- |
| **Number of symptoms** | **n** | **%** | **n** | **%** | **n** | **%** |
| 0 | 75 | 44.1 | 219 | 37.6 | 47725 | 79 |
| 1 | 28 | 16.5 | 107 | 18.4 | 6179 | 10.2 |
| 2 | 18 | 10.6 | 62 | 10.6 | 3056 | 5.1 |
| 3 | 10 | 5.9 | 46 | 7.9 | 1654 | 2.7 |
| 4 | 15 | 8.8 | 47 | 8.1 | 778 | 1.3 |
| 5 | 9 | 5.3 | 26 | 4.5 | 445 | 0.7 |
| 6 | 3 | 1.8 | 14 | 2.4 | 256 | 0.4 |
| 7 | 5 | 2.9 | 18 | 3.1 | 158 | 0.3 |
| 8 | 3 | 1.8 | 13 | 2.2 | 81 | 0.1 |
| 9 | 1 | 0.6 | 5 | 0.9 | 44 | 0.1 |
| 10+ | 3 | 1.8 | 26 | 4.5 | 58 | 0 |

^a^ Participants with symptom duration >6 months or >12 months were excluded according to time since initial infection.

^b^ Participants with symptom duration >12 months were excluded.

**Supplementary Table 7. Bivariate tetrachoric correlation coefficients between 20 symptoms among all COVID-19 cases (n=774). Rare occurrences (myocarditis and kidney disease) were excluded.**

|  | **Fatigue** | **Fever** | **Shortness of breath** | **Cough** | **Reduced lung function** | **Joint pain** | **Muscle pain** | **Chest pain** | **Heart palpitation** | **Dizziness** | **Brain fog** | **Poor memory** | **Mood changes** | **Sleep problems** | **Headache** | **Depression** | **Anxiety** | **Skin rash** | **Hair loss** | **Altered smell or taste** |
| --- | --- | --- | --- | --- | --- | --- | --- | --- | --- | --- | --- | --- | --- | --- | --- | --- | --- | --- | --- | --- |
| **Fatigue** | 1 | 0.17 | 0.64 | 0.33 | 0.37 | 0.42 | 0.41 | 0.49 | 0.65 | 0.59 | 0.7 | 0.73 | 0.59 | 0.52 | 0.61 | 0.51 | 0.49 | 0.38 | 0.56 | 0.33 |
| **Fever** | 0.17 | 1 | 0.09 | -0.36 | -0.38 | 0.36 | 0.38 | 0.36 | 0.23 | 0.39 | 0.34 | 0.23 | 0.22 | 0.05 | 0.42 | -0.33 | -0.36 | 0.27 | 0.22 | 0.25 |
| **Shortness of breath** | 0.64 | 0.09 | 1 | 0.47 | 0.58 | 0.46 | 0.48 | 0.56 | 0.45 | 0.38 | 0.45 | 0.57 | 0.32 | 0.38 | 0.54 | 0.24 | 0.3 | 0.35 | 0.32 | 0.25 |
| **Cough** | 0.33 | -0.36 | 0.47 | 1 | 0.38 | 0.02 | 0.22 | 0.18 | 0.18 | -0.01 | 0.12 | 0.19 | -0.04 | 0.26 | 0.23 | 0.07 | 0.12 | 0.17 | 0.27 | 0.15 |
| **Reduced lung function** | 0.37 | -0.38 | 0.58 | 0.38 | 1 | 0.12 | 0.03 | 0.4 | 0.2 | 0.26 | 0.31 | 0.37 | -0.05 | 0.05 | 0.23 | 0.1 | 0.27 | 0.09 | 0 | 0.13 |
| **Joint pain** | 0.42 | 0.36 | 0.46 | 0.02 | 0.12 | 1 | 0.67 | 0.38 | 0.51 | 0.4 | 0.41 | 0.4 | 0.37 | 0.36 | 0.44 | 0.24 | 0.33 | 0.36 | 0.18 | 0.21 |
| **Muscle pain** | 0.41 | 0.38 | 0.48 | 0.22 | 0.03 | 0.67 | 1 | 0.4 | 0.49 | 0.57 | 0.46 | 0.5 | 0.43 | 0.46 | 0.5 | 0.22 | 0.25 | 0.34 | 0.39 | 0.25 |
| **Chest pain** | 0.49 | 0.36 | 0.56 | 0.18 | 0.4 | 0.38 | 0.4 | 1 | 0.35 | 0.4 | 0.45 | 0.46 | 0.11 | 0.24 | 0.52 | -0.05 | 0.16 | 0.08 | 0.41 | 0.05 |
| **Heart palpitation** | 0.65 | 0.23 | 0.45 | 0.18 | 0.2 | 0.51 | 0.49 | 0.35 | 1 | 0.66 | 0.63 | 0.65 | 0.54 | 0.61 | 0.57 | 0.4 | 0.44 | 0.28 | 0.49 | 0.31 |
| **Dizziness** | 0.59 | 0.39 | 0.38 | -0.01 | 0.26 | 0.4 | 0.57 | 0.4 | 0.66 | 1 | 0.65 | 0.63 | 0.51 | 0.55 | 0.65 | 0.27 | 0.3 | 0.41 | 0.4 | 0.24 |
| **Brain fog** | 0.7 | 0.34 | 0.45 | 0.12 | 0.31 | 0.41 | 0.46 | 0.45 | 0.63 | 0.65 | 1 | 0.84 | 0.58 | 0.6 | 0.6 | 0.43 | 0.33 | 0.48 | 0.41 | 0.27 |
| **Poor memory** | 0.73 | 0.23 | 0.57 | 0.19 | 0.37 | 0.4 | 0.5 | 0.46 | 0.65 | 0.63 | 0.84 | 1 | 0.45 | 0.54 | 0.52 | 0.41 | 0.39 | 0.38 | 0.44 | 0.41 |
| **Mood changes** | 0.59 | 0.22 | 0.32 | -0.04 | -0.05 | 0.37 | 0.43 | 0.11 | 0.54 | 0.51 | 0.58 | 0.45 | 1 | 0.62 | 0.66 | 0.77 | 0.67 | 0.55 | 0.52 | 0.17 |
| **Sleep problems** | 0.52 | 0.05 | 0.38 | 0.26 | 0.05 | 0.36 | 0.46 | 0.24 | 0.61 | 0.55 | 0.6 | 0.54 | 0.62 | 1 | 0.51 | 0.56 | 0.45 | 0.15 | 0.46 | 0.2 |
| **Headache** | 0.61 | 0.42 | 0.54 | 0.23 | 0.23 | 0.44 | 0.5 | 0.52 | 0.57 | 0.65 | 0.6 | 0.52 | 0.66 | 0.51 | 1 | 0.32 | 0.3 | 0.51 | 0.3 | 0.28 |
| **Depression** | 0.51 | -0.33 | 0.24 | 0.07 | 0.1 | 0.24 | 0.22 | -0.05 | 0.4 | 0.27 | 0.43 | 0.41 | 0.77 | 0.56 | 0.32 | 1 | 0.88 | 0.4 | 0.28 | 0.02 |
| **Anxiety** | 0.49 | -0.36 | 0.3 | 0.12 | 0.27 | 0.33 | 0.25 | 0.16 | 0.44 | 0.3 | 0.33 | 0.39 | 0.67 | 0.45 | 0.3 | 0.88 | 1 | 0.39 | 0.46 | 0.07 |
| **Skin rash** | 0.38 | 0.27 | 0.35 | 0.17 | 0.09 | 0.36 | 0.34 | 0.08 | 0.28 | 0.41 | 0.48 | 0.38 | 0.55 | 0.15 | 0.51 | 0.4 | 0.39 | 1 | 0.21 | 0.33 |
| **Hair loss** | 0.56 | 0.22 | 0.32 | 0.27 | 0 | 0.18 | 0.39 | 0.41 | 0.49 | 0.4 | 0.41 | 0.44 | 0.52 | 0.46 | 0.3 | 0.28 | 0.46 | 0.21 | 1 | 0.2 |
| **Altered smell or taste** | 0.33 | 0.25 | 0.25 | 0.15 | 0.13 | 0.21 | 0.25 | 0.05 | 0.31 | 0.24 | 0.27 | 0.41 | 0.17 | 0.2 | 0.28 | 0.02 | 0.07 | 0.33 | 0.2 | 1 |


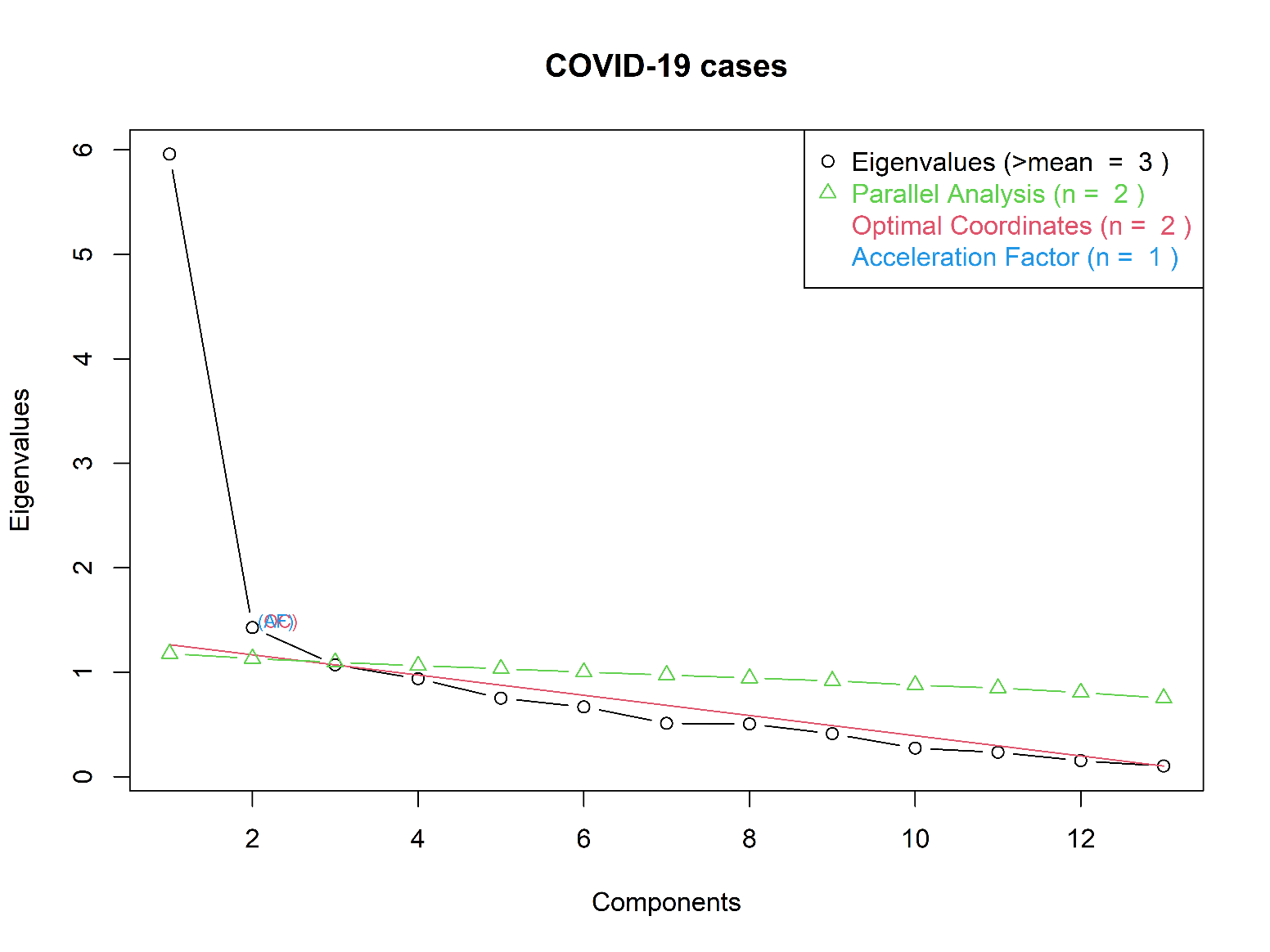


Supplementary Figure 1. Scree plot from R package *nFactors*. The first three factors had eigenvalues 6.0, 1.4 and 1.1. The Kaiser criterion (eigenvalue >1) suggested three factors and Horn’s parallel analysis suggested that two factors should be retained in the model. Parallel analysis was run with 100 replications of the correlation matrix.


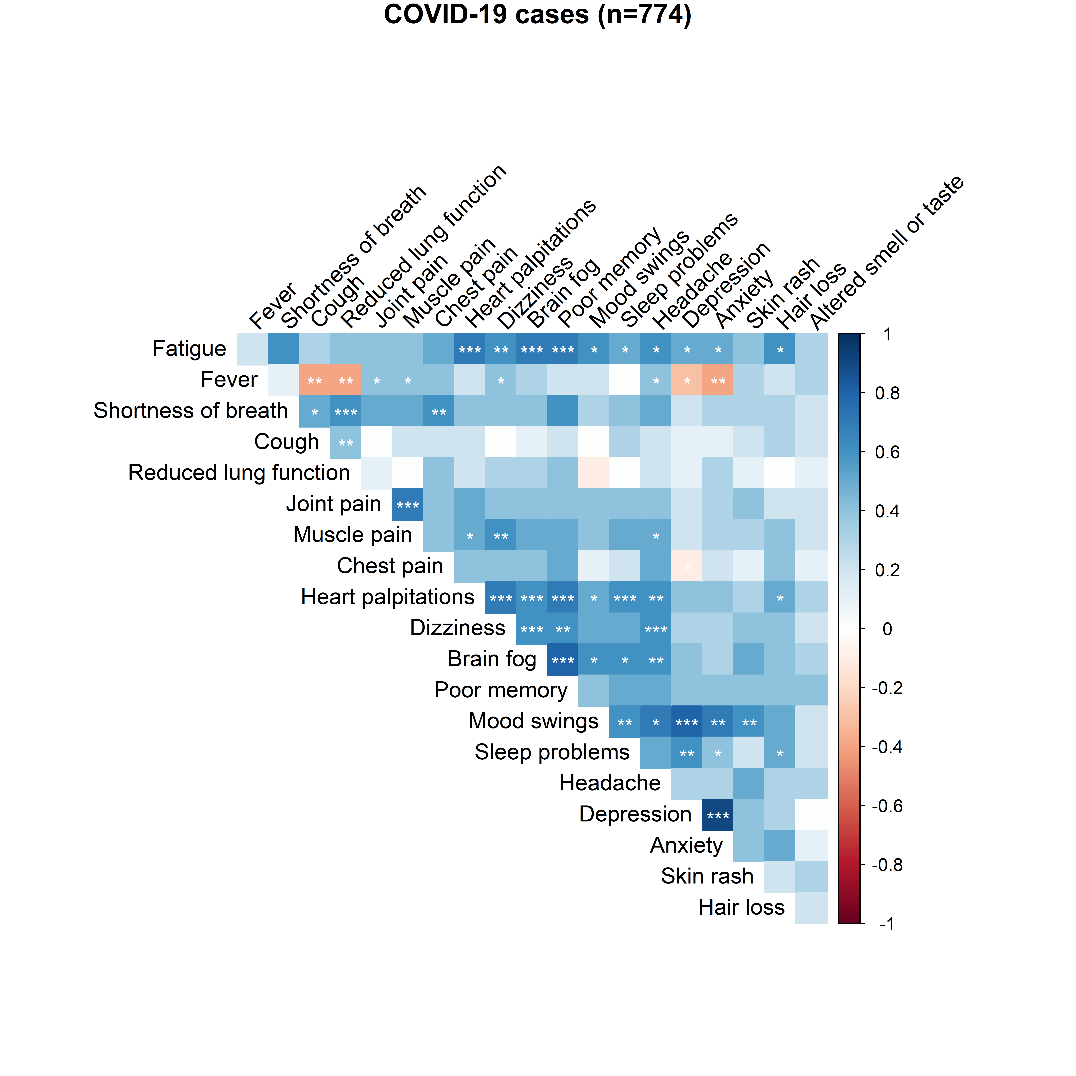


Supplementary Figure 2. Bivariate tetrachoric correlations between symptoms reported in March 2021 among COVID-19 cases (n=774). Rare occurrences (myocarditis and kidney disease) were excluded. The strength of correlation coefficients is indicated by the colour panel (right). Intensity of red colours indicate increasing negative correlation coefficients, while intensity of blue colours indicates increasing positive correlation coefficients. Asterisks indicating significant correlations (*** for p<.001; ** for p<.01; * for p<.05).
